# Atypical postural control can be detected via computer vision analysis in toddlers with autism spectrum disorder

**DOI:** 10.1038/s41598-018-35215-8

**Published:** 2018-11-19

**Authors:** Geraldine Dawson, Kathleen Campbell, Jordan Hashemi, Steven J. Lippmann, Valerie Smith, Kimberly Carpenter, Helen Egger, Steven Espinosa, Saritha Vermeer, Jeffrey Baker, Guillermo Sapiro

**Affiliations:** 1 Duke Center for Autism and Brain Development, Department of Psychiatry and Behavioral Sciences, Duke University, Durham, North Carolina USA; 2 University of Utah, Salt Lake City, Utah USA; 3 Department of Electrical and Computer Engineering, Duke University, Durham, North Carolina USA; 4 Department of Population Health Sciences, Duke University, Durham, North Carolina USA; 5 NYU Langone Child Study Center, New York University, New York, New York, USA; 6 Department of Pediatrics, Duke University, Durham, NC USA; 7 Departments of Biomedical Engineering, Computer Science, and Mathematics, Duke University, Durham, NC USA

**Keywords:** Paediatric research, Risk factors, Disability

## Abstract

Evidence suggests that differences in motor function are an early feature of autism spectrum disorder (ASD). One aspect of motor ability that develops during childhood is postural control, reflected in the ability to maintain a steady head and body position without excessive sway. Observational studies have documented differences in postural control in older children with ASD. The present study used computer vision analysis to assess midline head postural control, as reflected in the rate of spontaneous head movements during states of active attention, in 104 toddlers between 16–31 months of age (Mean = 22 months), 22 of whom were diagnosed with ASD. Time-series data revealed robust group differences in the rate of head movements while the toddlers watched movies depicting social and nonsocial stimuli. Toddlers with ASD exhibited a significantly higher rate of head movement as compared to non-ASD toddlers, suggesting difficulties in maintaining midline position of the head while engaging attentional systems. The use of digital phenotyping approaches, such as computer vision analysis, to quantify variation in early motor behaviors will allow for more precise, objective, and quantitative characterization of early motor signatures and potentially provide new automated methods for early autism risk identification.

## Introduction

Although the core symptoms of autism spectrum disorder (ASD) are defined by atypical patterns of social interaction and the presence of stereotyped and repetitive behaviors and interests, evidence suggests that differences in motor function are also an important early feature of autism. Motor delays could contribute to early hallmark autism symptoms, including difficulties in orienting to name involving the eyes and head turns, coordinating head and limb movements involved in gaze following and other joint attention behavior, such as pointing. Teitelbaum *et al*.^1^ found that atypical movements (e.g. shape of mouth, patterns of lying, righting, sitting) were present by 4–6 months of age in infants later diagnosed with ASD. Another study of videotapes taken of infants 12–21 weeks of age detected lower levels of positional symmetry among infants later diagnosed with ASD^2^ suggesting atypical development of cerebellar pathways that control balance and symmetry. Six-month-old infants who later were diagnosed with ASD tend to exhibit head lag when pulled to sit, reflecting early differences in motor development^3^. A study of home videos taken between birth and six months of age found that some infants who were later diagnosed with ASD showed postural stiffness, slumped posture, and/or head lag^4^. Other motor symptoms observed in infants later diagnosed with ASD include fluctuating muscle tone^5^ and oral-motor abnormalities, such as insufficient opening of the mouth in anticipation of the approaching spoon during feeding^6^. Longitudinal research with very low birth weight infants revealed that infants who are later diagnosed with ASD had poorer ability in maintaining midline position of the head at 9–20 weeks of age^7^. The authors used visual inspection to classify head position in each video frame to yield a measure of midline head position and number of changes in position.

The development of postural control is an index of neuromuscular reactions to the motion of body mass in order to retain stability. Previous studies have documented the developmental progression of the ability to maintain an upright posture that is accompanied by decreases in postural sway^8^. Several studies with older children with ASD have documented deficiencies in postural control, reflected in the presence of postural sway, which is accentuated when children with ASD are viewing arousing stimuli, including complex multi-sensory and social stimuli^9–11^. Less is known about the presence of postural sway in young children with ASD.

Studies of motor and other behaviors in young children have typically relied on subjective and labor-intensive human coding to rate and measure behavior. The recent use of digital phenotyping approaches, such as computer vision analysis (CVA) of videotaped recordings of behavior, has allowed for automated, precise and quantitative measurement of subtle, dynamic differences in motor behavior. We reported previously on a result using CVA to more precisely measure toddlers’ orienting response to a name call, noting that, compared to toddlers without ASD, toddlers with ASD oriented less frequently; when they did orient, their head turn was a full second slower, on average^12^. Such differences in motor speed would likely not be detected with the naked eye during a typical clinical evaluation. Anzulewicz *et al*.^13^ used smart tablet computers with touch-sensitive screens and embedded inertial movement sensors to record movement kinematics and gesture forces in 3–6-year-old children with and without ASD. Children with ASD used greater force and faster and larger gesture kinematics. Machine learning analysis of the children’s motor patterns classified the children with ASD with a high level of accuracy. In another study using automated methods, differences in head movement dynamics were found between 2.5–6.5-year-old children with and without ASD while they watched movies of social and nonsocial stimuli. Children with ASD showed more frequent head turning, especially while watching social stimuli^14^. The authors suggested that the children with ASD might be using head movement to modulate their arousal while watching social stimuli. Wu *et al*.^15^ used electromagnetic sensors to analyze continuous movements at millisecond time scale in older children with ASD versus typical development. They applied a triangular smoothing algorithm to the 3D positional raw movement data that preserved the local speed fluctuations. They found that individuals with ASD exhibited significantly more “sensorimotor noise” when compared to individuals with typical development.

The present study used CVA to characterize head movements that didn’t involve spontaneous or volitional orienting or turning away from the stimuli. Rather, we were interested in subtler midline head movements that are more likely related to postural stability. The study compared the behaviors of toddlers with ASD versus those without ASD while the children watched a series of dynamic movies involving different types of stimuli, including stimuli of both a social and nonsocial nature. While the children watched the movies, their head movements were automatically detected and tracked using landmarks on the participant’s face. The goal of this analysis was to quantify the rate of spontaneous head movements and to determine whether there were differences in this motor feature between young children with and without ASD.

## Methods

### Participants

Participants were 104 children between 16–31 months of age (Mean = 22 months). Exclusionary criteria included known vision or hearing deficits, lack of exposure to English at home and/or caregivers who did not speak and read English sufficiently for informed consent. Twenty-two of the children had autism spectrum disorder. The non-ASD comparison group was comprised of 96 typically developing children and 8 children with language delay or developmental delay of clinical significance sufficient to qualify for speech or developmental therapy. Participants in the comparison group had a mean age of 21.91 months (SD = 3.78) and those in the ASD group had a mean age of 26.19 months (SD = 4.07). Ethnic/racial composition of the ASD and comparison groups, respectively, was 59% and 45% white, 13% and 14% African American, 6% and 5% Asian, and 22% and 36% multi-racial/other. Percent males was 77% in the ASD group and 59% in the comparison group.

Participants were recruited from primary care pediatric clinics by a research assistant, referral from their physician, and by community advertisement. All caregivers/legal guardians of participants gave written, informed consent, and the study protocol was approved by the Duke University Health System Institutional Review Board. Methods were carried out in accordance with institutional, state, and federal guidelines and regulation.

### Diagnostic Assessments

Diagnostic evaluations to confirm ASD were based on the Autism Diagnostic Observation Scale-Toddler (ADOS-T), which were conducted by a licensed psychologist or trained research-reliable examiner overseen by a licensed psychologist^16^. The mean ADOS-T score was 18.81 (SD = 4.20). The mean IQ based on the Mullen Scales of Early Learning Composite Score for the ASD group was 63.58 (SD = 25.95). Developmental and/or language delay was determined based on the Mullen Scales (>1 SD below the mean in overall learning composite or receptive/expressive language).

### Stimuli

**Figure 1:**
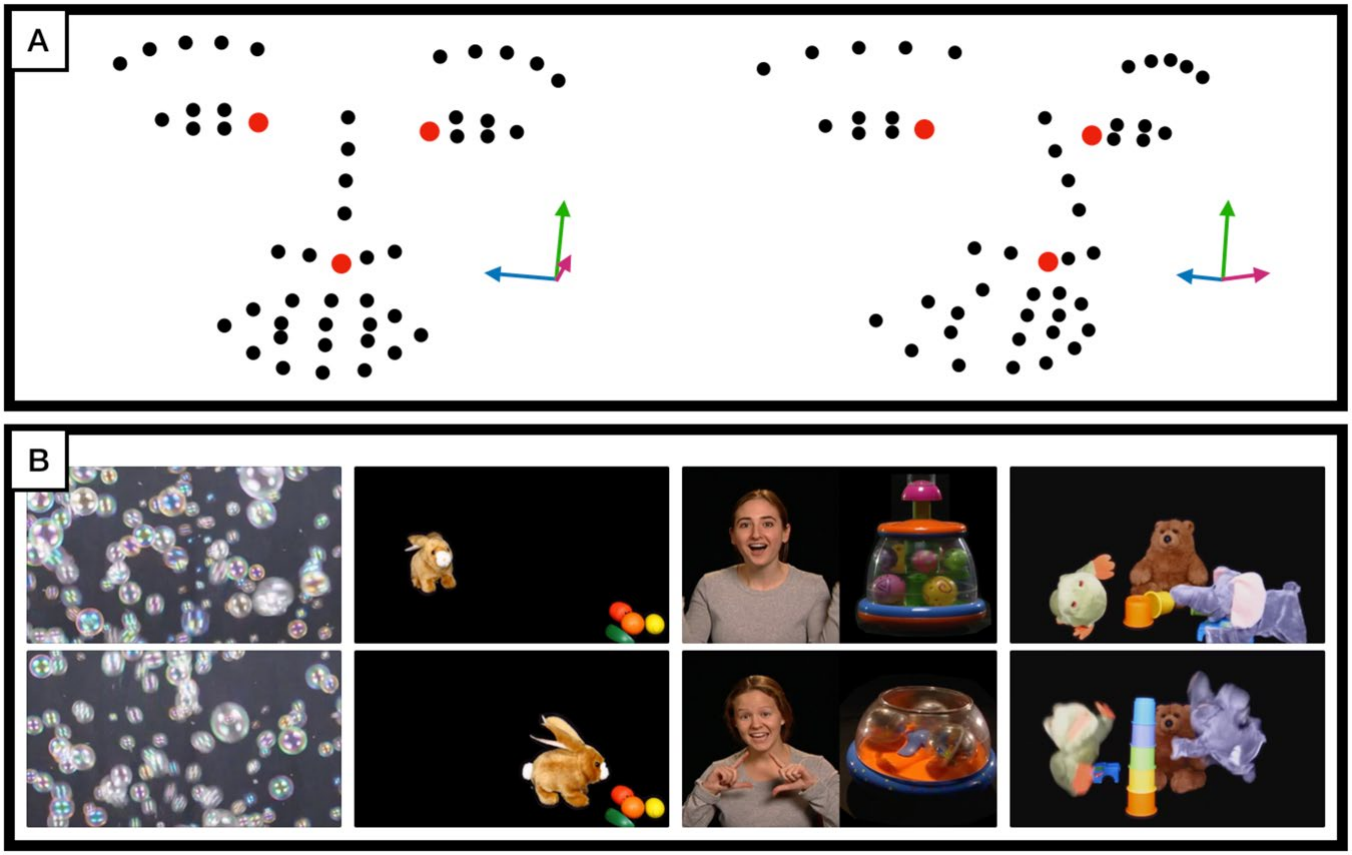
iPad movie task and facial landmark detection: (**A**) Two examples of facial landmark points detected by CVA and estimated head pose (indicated by the three arrows). The landmarks colored in red are the inner left, inner right, and central nose landmarks that are used for head movement computation. The left example depicts landmarks and head pose of a participant engaged in the movie stimuli; while in the right example, the participant is looking away. Both states are automatically detected. (**B**) Example frames from movie stimuli. Each row displays a frame from corresponding movie stimuli show in the columns (going from left-to-right): Bubbles (30 seconds, two repetitions), Bunny (66 seconds), Rhymes (60 seconds), and Puppet show (68 seconds).

A series of stimuli, comprised of brief movies, were shown on a smart tablet while the child sat on a caregiver’s lap. The tablet was placed on a stand approximately 3 feet away from the child to prevent the child from touching the screen. The stimuli consisted of a series of brief developmentally-appropriate movies designed to elicit positive affect and engage the child’s attention. The movies consisted of cascading bubbles, a mechanical bunny, animal puppets interacting with each other, and a split screen showing on one side a woman singing nursery rhymes and on the other side dynamic, noise-making toys. The lengths of the movies were 30 seconds (Bubbles), 60 seconds (Rhyme), and ∼70 seconds (Bunny and Puppets). Each movie was shown once except for Bubbles which was shown at the beginning and end of the series. The entire series of movies lasted 5 minutes. Examples of the stimuli and experimental setup are presented in Fig. 1 and described in two previous publications^17^. Examples of clips from the movies are provided in the Supplementary Material. During three of the movies, the examiner, standing behind the child, called the child’s name. A failure to orient to name is an early symptom of autism, and results of our analysis of the orienting results have previously been published^12^. However, all segments when children looked away from the movie, including to orient to name, as well as all 5 second segments post the name-call stimulus, were automatically removed from the present analyses. Specifically, in order to remove any influence on head movement due to the child orienting when his or her name was called, we removed the time window starting at cue for the name call prompt (a subtle icon used to prompt the examiner to call the name) through the point where 75% of the audible name calls actually occurred, plus 150 frames (5 seconds). Since previous studies have shown that orienting tends to occur within a few seconds after a name call, this eliminated segments influenced by the name call.

Parents were asked to attempt to keep the child seated in their lap, but to allow the child to get off their lap if the child became too distressed to stay seated. Researchers stopped the task for 1 child due to crying. Researchers restarted the task for three participants due to noncompliance.

### Computer Vision Analysis

The frontal camera in the tablet recorded video of the child’s face throughout the experiment at 1280 × 720 spatial resolution and 30 frames per second. The fully automatic CVA algorithm detects and tracks 49 facial landmarks on the child’s face (see Fig. 1)^18^ and estimates head pose angles relative to the camera by computing the optimal rotation parameters between the detected landmarks and a 3D canonical face model^19^. For each video frame the algorithm outputted 2D positional coordinates of the facial landmarks and 3 head pose angles: yaw (left-right), pitch (up-down), and roll (tilting left-right). The yaw head pose angle was used to determine the frames when the child was engaged with the movie stimuli, where frames exhibiting a yaw pose with a magnitude less than 20° were considered as the child being engaged.

Following the work of^17^, to quantify head movement when the child is engaged (less than 20° yaw), per-frame pixel-wise displacements of 3 central facial landmarks were computed and normalized with respect to the child’s eye width, thus head movement was measured as a (normalized) proportion of the child’s eye width per frame. The pixel-wise displacements of the central facial landmarks are dependent on the child’s distance to the camera in the tablet. Although the tablet was placed approximately 3 feet away from the child at the start of the experiment, the child is free to move throughout the experiment, thus affecting the magnitude of landmark displacements (when the child is near to the camera the pixel displacements are larger than if the child did the same movement but farther away from the camera). Normalizing the displacements with respect to the eye-width diminishes this distance to camera dependency. More formally, the head movement between frame *n* and *n-1* is defined as the average Euclidean displacements of the central nose, left inner eye, and right inner eye landmarks (see Fig. 1) normalized by a ± second windowed-average, centered around frame *n*, of the Euclidean distances between the inner left and right eye landmarks,

$$\frac{\overline{{d}_{n-1,n}}}{\overline{{w}_{n-15,n+15}}}\,,$$

where $\overline{{d}_{n-1,n}}$ is the average landmark displacement of the three central landmarks between frame *n* and *n-1*, and $\overline{{w}_{n-15,n+15}}$ is the average Euclidean distance between the left and right eye landmarks when the child is engaged between a half-second (15 frames) before and after frame *n*.

Results evaluating the validity of the CVA methods, which rely on landmark identification and tracking on the face, have been previously published. One study demonstrated high reliability between the automatic methods and the expert human rater of head movements, with agreement between the computer and expert clinical rater occurring 92.5% of the time with interrater reliability based on Cohen’s kappa = 0.75^20^. A second study compared the automatic classification based on landmarks to human coders for head movement, demonstrated inter-rater reliability based on intraclass correlation coefficient (ICC) = 0.89^17^. Other papers report high reliability between CVA and human coding for head turning in response to name (ICC = 0.84)^12^ and positive affective expression (happy; ICC = 0.90 and 0.89 for ASD and non-ASD toddlers)^21^.

The original dataset consisted of frame-by-frame measurements of head movement, with observations for each 1/30^th^ of a second. Groups interested in direct use of the data can do so via collaboration with the authors due to privacy and consent considerations as well as backend designs, and the data will be stored in a separate partition at Duke University. In order to prepare the data for statistical analysis, we first aggregated the movement measurements by calculating the head movement rate, defined as the moving sum of the cumulative frame-by-frame movement measurements for each 10 frame period (representing 1/3^rd^ of a second). If any individual frames within a 10-frame set were set to missing, such as when the facial landmarks were not visible or during the name-call period, the moving sum was also set to missing. Outliers were addressed by Winsorizing to the 95^th^ percentile prior to aggregation.

All statistical analyses were performed separately for each of the movie stimuli. To visualize the time series, we calculated and plotted the median head movement rate as well as the 1^st^ and 3^rd^ quartiles at each 1/3 second time interval for both ASD and non-ASD children.

Unadjusted and adjusted rate ratios for the association between ASD diagnosis and the rate of head movement in each 1/3 second time interval were estimated using a generalized linear mixed log-gamma regression model. Adjusted estimates controlled for ethnicity/race (white; other), age (in months), and sex (male; female). To account for potential within-subject correlations due to repeated measurement, we included a random intercept for each participant.

## Results

**Figure 2:**
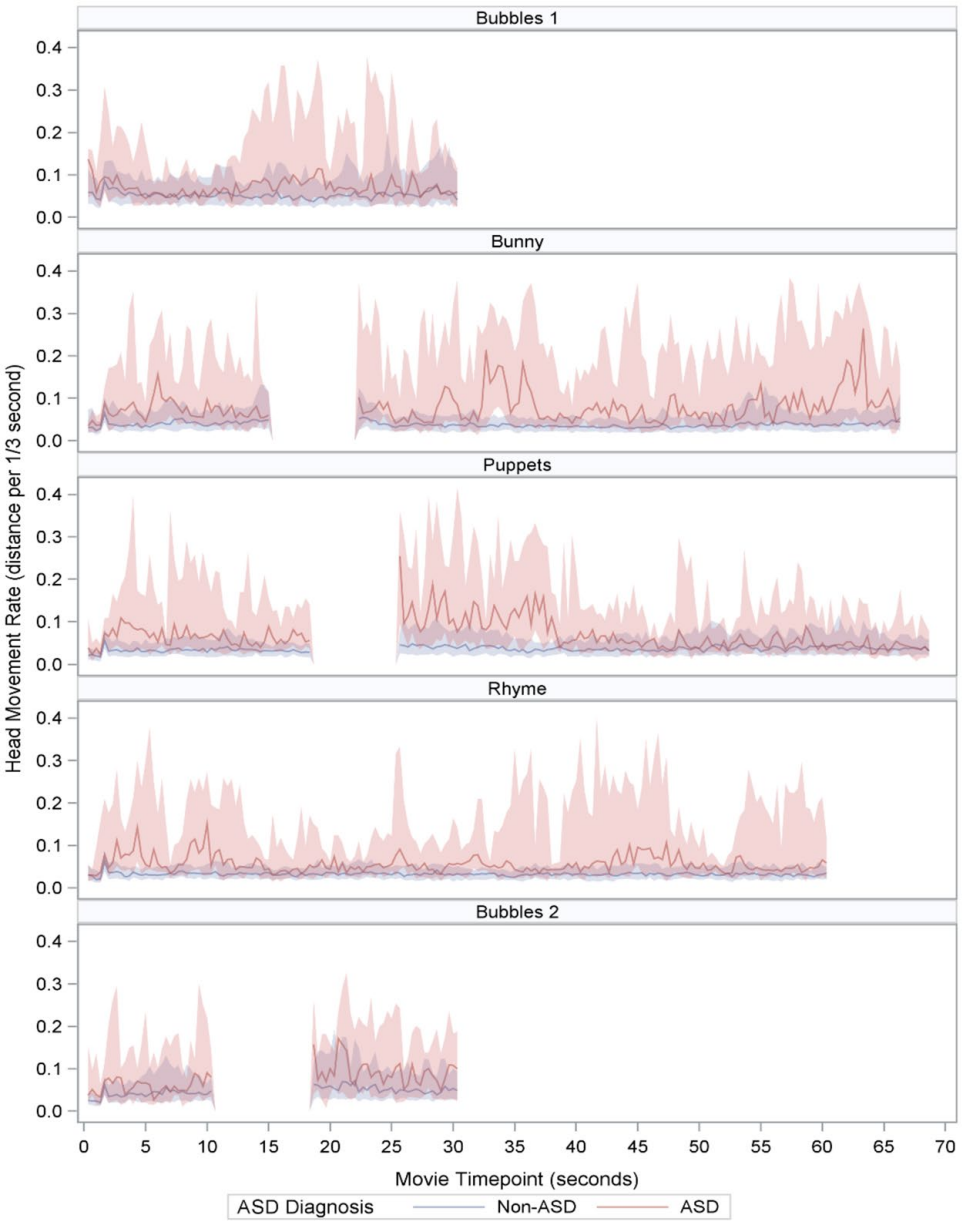
Time series of head movement rate, measured as the distance traveled per 1/3 seconds (10 video frames), by ASD diagnosis. Solid lines are the median values at each time point. Bands represent the first and third quartiles at each time point. Blank sections represent name calls, which were removed from this analysis.

The time series data depicting the rate of head movement, defined as the distance traveled per 1/3 second (10 videoframes), for the ASD and non-ASD groups are shown in Fig. 2.

Based on a generalized linear mixed regression model with a log link and gamma distribution (adjusting for ethnicity/race, age, and sex), significant associations between diagnostic group (ASD versus non-ASD) and rate of head movement were found during all movies except for the Bubbles 2, the last movie. For Bubbles 2, the shorter duration might have affected power to detect a result as there was nevertheless a trend toward a group difference in the same direction as all other movies. Results of the analysis are shown in Table 1.

**Table 1:** Unadjusted and adjusted rate ratios for the associations between diagnostic group and rate of head movement.

Movie	Unadjusted		Adjusted	
	Rate Ratio (95% Confidence Interval) for ASD vs non-ASD	P-value	Rate Ratio (95% Confidence Interval) for ASD vs non-ASD	P-value
Video Bubbles 1	1.46 (1.09, 1.97)	0.011	1.53 (1.10, 2.12)	0.012
Video Bunny	2.13 (1.60, 2.85)	<0.0001	2.22 (1.60, 3.07)	<0.0001
Video Puppets	2.08 (1.50, 2.88)	<0.0001	2.30 (1.60, 3.31)	<0.0001
Video Rhymes and Toys	2.37 (1.77, 3.16)	<0.0001	2.45 (1.78, 3.39)	<0.0001
Video Bubbles 2	1.52 (1.08, 2.14)	0.018	1.43 (0.97, 2.10)	0.070

Robust group differences in the rate of head movement were evident during 4 out of 5 of the movies. For example, the rate of head movement among participants with ASD was 2.22 times that of non-ASD participants during the Bunny movie, after adjusting for age, ethnicity/race, and sex (95% Confidence Interval 1.60, 3.07). The rate ratio was higher for all movies that had animated and more complex stimuli (Bunny, Puppets, Rhymes and Toys), as compared to the less complex Bubbles videos.

Although the LD/DD group was too small to conduct independent analyses of that group, as a sensitivity analysis, the 8 patients with LD/DD were from the main regression model and re-estimated the associations, as shown in Table 2. Overall, the results are consistent with those reported in the main analysis; in fact, the associations are slightly stronger when the LD/DD group is removed from the non-ASD group.

**Table 2:** Adjusted rate ratios for the associations between diagnostic group and rate of head movement after removing LD/DD participants.

Movie	Adjusted Rate Ratio (95% Confidence Interval) for ASD vs TD	P-value
Video Bubbles 1	1.58 (1.11, 2.24)	0.0109
Video Bunny	2.34 (1.67, 3.30)	<0.0001
Video Puppets	2.38 (1.62, 3.50)	<0.0001
Video Rhymes and Toys	2.54 (1.81, 3.57)	<0.0001
Video Bubbles 2	1.50 (1.00, 2.26)	0.0496

## Discussion

The present study adds to a large and growing body of literature indicating that differences in early motor development are an important feature of ASD. We found highly significant differences in postural control, reflected in differences in the rate of spontaneous movement of the head between toddlers with ASD versus those without ASD. Using an automated, objective approach, we analyzed data comprised of video-frame-level measurements of head movements with observation for each 1/30^th^ of a second and created 10-frame moving sums to capture movement. Time-series data revealed group differences in the rate of head movement across all movies representing a wide range of stimuli, such as bubbles, a hopping bunny, and a woman singing a nursery rhyme paired with dynamic toys. An increase in the rate of head movement observed in young children with ASD during states of engaged attention might indicate underlying differences in the ability to maintain midline postural control and/or atypical engagement of attentional systems in young toddlers with ASD. These movements were not defined by spontaneous looking away from the stimulus, as was reported by Martin *et al*.^14^. Rather, they were characterized by a failure to keep the head in a still midline position while viewing the movie. This is distinct from the feature studied by Martin *et al*., which was characterized by greater yaw angular displacement and greater yaw and roll angular velocity, which was primarily present during the presentation of social stimuli and might reflect sensory modulation. The movements we describe in this paper may be similar to those described in previous studies of postural sway in older children with ASD, as well as school aged children with attention deficit hyperactivity disorder (ADHD) by Heiser *et al*.^22^. Heiser *et al*. used infrared motion analysis to record head movements during a continuous performance task and found that boys with ADHD moved their head 2.3 times as far as typically-developing boys performing the same task. In a study of siblings of children with ASD, Reiersen and colleagues^23^ found that siblings who have impaired motor coordination, features of attention deficit hyperactivity disorder (ADHD), or both are much more likely to have ASD than are other siblings. They suggest that identification of nonspecific traits that can amplify risk for ASD, such as attention and motor differences, could allow for earlier identification and targeted therapy that modify these traits and potentially reduce later risk for ASD.

Delays and differences in sensorimotor development have been noted across the lifespan in individuals with ASD from early infancy through adulthood^24^. For example, Lim *et al*. showed that postural sway and attention demands of postural control were larger in adults with ASD than in typically developed adults^25^. Morris *et al*. found that adults with ASD did not use visual information to control standing posture, in contrast to adults without ASD^26^. Brain imaging studies suggest that atypical motor function in autism may be related to increased sensitivity to proprioceptive error and a decreased sensitivity to visual error, aspects of motor learning dependent on the cerebellum^27^. Atypical presentation of motor functions of the cerebellum has been noted in children with ASD as young as 14 months of age. Esposito *et al*. identified significant differences in gait pattern, reflected in postural asymmetry, in toddlers with ASD as compared to those without ASD^28^.

The sample of toddlers with ASD was recruited from primary pediatric care where children suspected of having autism were then evaluated using gold-standard diagnostic methods. Although this method of recruitment increases the likelihood of obtaining a more representative population-based sample, it also results in a comparison group of toddlers without ASD that is much larger than the ASD sample. Because the sample of ASD toddlers in this study was relatively small, it will be important to replicate these findings with a larger group of children. A larger sample would also provide the statistical power to examine whether differences in postural control exist based on individual characteristics of children with ASD, such as age, sex, and co-morbid intellectual disability and/or ADHD.

Previous analyses of motor differences associated with ASD often have required labor-intensive coding of patterns of behavior that are recognizable by the naked eye. Moreover, such studies typically use a “top down” approach in which specific behaviors of interest are defined and then rated by more than one person (for reliability assessments). The use of digital phenotyping offers multiple advantages over previous methods that rely on human coding, namely, the ability to automatically and objectively measure dynamic features of behavior on a spatiotemporal scale that is not easily perceptible to the naked eye. Because digital approaches are scalable, they also allow for collection of larger data sets that can be analyzed using machine learning. We anticipate that the use of digital phenotyping will reveal a number of objective biomarkers, such as the head movements described in this report, which can be used as early risk indices and targets for intervention. By combining multiple features that reflect different aspects of sensorimotor function, including patterns of facial expressiomn, orienting, midline head movements, reaching behavior, and others, it might be possible to create a reliable, objective and automated risk profile for ASD and other neurodevelopmental disorders.

## Electronic supplementary material

Supplementary Material Information

Movie Stimuli
