# Author Correction: Atypical postural control can be detected via computer vision analysis in toddlers with autism spectrum disorder

**DOI:** 10.1038/s41598-020-57570-1

**Published:** 2020-01-14

**Authors:** Geraldine Dawson, Kathleen Campbell, Jordan Hashemi, Steven J. Lippmann, Valerie Smith, Kimberly Carpenter, Helen Egger, Steven Espinosa, Saritha Vermeer, Jeffrey Baker, Guillermo Sapiro

**Affiliations:** 1 Duke Center for Autism and Brain Development, Department of Psychiatry and Behavioral Sciences, Duke University, Durham, North Carolina USA; 2 University of Utah, Salt Lake City, Utah USA; 3 Department of Electrical and Computer Engineering, Duke University, Durham, North Carolina USA; 4 Department of Population Health Sciences, Duke University, Durham, North Carolina USA; 5 NYU Langone Child Study Center, New York University, New York, New York USA; 6 Department of Pediatrics, Duke University, Durham, NC USA; 7 Departments of Biomedical Engineering, Computer Science, and Mathematics, Duke University, Durham, NC USA

Correction to: *Scientific Reports* 10.1038/s41598-018-35215-8, published online 19 November 2018

This Article contains errors in the Methods section under subheading ‘Diagnostic Assessments’.

“The mean ADOS-T score was 18.81 (SD = 4.20).”

should read:

“The mean ADOS-T score was 18.00 (SD = 4.67).”

