# Supplementary material for: Atypical postural control can be detected via computer vision analysis in toddlers with autism spectrum disorder
Source: Sci Rep. 2018 Nov 19;8:17008. doi: 10.1038/s41598-018-35215-8 (PMC6242931; doi:10.1038/s41598-018-35215-8)
Supplement: Supplementary file 1 — Supplementary Material Information [file 41598_2018_35215_MOESM1_ESM.docx]

Supplementary Material: Examples of Movie Stimuli

Atypical postural control can be detected via computer vision analysis in toddlers

with autism spectrum disorder

Geraldine Dawson^1^, Kathleen Campbell^2^, Jordan Hashemi^1,3^, Steven J. Lippmann^4^, Valerie Smith^4^, Kimberly Carpenter^1^, Helen Egger^5^, Steven Espinosa^3^, Saritha Vermeer^1^, Jeffrey Baker^6^, and Guillermo Sapiro^3,7^

^1^ Duke Center for Autism and Brain Development, Department of Psychiatry and Behavioral Sciences, Duke University, Durham, North Carolina

^2^ University of Utah, Salt Lake City, Utah

^3^ Department of Electrical and Computer Engineering, Duke University, Durham, North Carolina

^4^ Department of Population Health Sciences, Duke University, Durham, North Carolina

^5^ NYU Langone Child Study Center, New York University, New York, New York

^6^ Department of Pediatrics, Duke University, Durham, NC

^7^ Departments of Biomedical Engineering, Computer Science, and Mathematics, Duke University, Durham, NC

Corresponding author: Geraldine Dawson

Duke University, Duke Center for Autism and Brain Development, 2608 Erwin Road, Suite 300, Durham, NC, 27705

Email: [geraldine.dawson@duke.edu](mailto:geraldine.dawson@duke.edu)
